# Eye-Tracking Technologies for Cognitive Assessment After Acquired Brain Injury: Systematic Review

**DOI:** 10.2196/81276

**Published:** 2026-06-02

**Authors:** Andrea Calderone, Rosaria De Luca, Francesco Corallo, Rosalia Calapai, Alessio Mirabile, Angelo Quartarone, Alessandro Marco De Nunzio, Carmela Casella, Rocco Salvatore Calabrò

**Affiliations:** 1 Department of Neurorehabilitation, IRCCS Centro Neurolesi Bonino-Pulejo, S.S. 113 Via Palermo, C.da Casazza, Messina, 98124, Italy; 2 Department of Health, LUNEX University of Applied Sciences, LUNEX University of Applied Sciences, 50 Avenue du Parc des Sports, Differdange, 4671, Luxembourg; 3 Luxembourg Health & Sport Sciences Research Institute, 50 Avenue du Parc des SportsDifferdange, 4671, Luxembourg; 4 Stroke Unit, University of Messina, Piazza Pugliatti 1, Messina, 98122, Italy

**Keywords:** acquired brain injury, eye-tracking technology, cognitive assessment, oculomotor biomarkers, saccadic eye movements, traumatic brain injury, neurocognitive impairment, neurorehabilitation

## Abstract

**Background:**

Acquired brain injury (ABI) is a heterogeneous umbrella term encompassing traumatic and nontraumatic etiologies and is frequently associated with persistent cognitive dysfunction. Conventional neuropsychological assessment remains central to clinical evaluation, but feasibility and measurement precision may be limited in individuals with motor impairment, aphasia, reduced stamina, or fluctuating arousal. Eye tracking offers an objective, low-burden approach that can quantify gaze behavior during task engagement and may provide complementary process-level markers of cognition.

**Objective:**

This study aimed to systematically synthesize the evidence on eye-tracking paradigms used as a primary approach for cognitive assessment in ABI and to summarize findings by cognitive domain, paradigm, and clinical interpretability.

**Methods:**

We conducted a PRISMA (Preferred Reporting Items for Systematic Reviews and Meta-Analyses) 2020–compliant systematic review and registered the protocol in PROSPERO (CRD420251038768). PubMed, Web of Science, the Cochrane Library, Embase, EBSCOhost, PsycINFO, and Scopus were searched from inception to April 10, 2025. We included peer-reviewed English-language studies enrolling children or adults with ABI in which eye tracking was the primary assessment modality used to quantify at least one cognitive domain or clinically relevant cognitive-communication process. Two reviewers independently screened studies, extracted data, and assessed methodological quality using design-appropriate tools (Risk of Bias 2, Risk of Bias in Non-Randomized Studies of Interventions, Quality Assessment of Diagnostic Accuracy Studies 2, and the Newcastle-Ottawa Scale). A structured narrative synthesis was performed because of heterogeneity in paradigms and outcome definitions.

**Results:**

Twenty-seven studies met the inclusion criteria (N=872 participants; females: n=354 and males: n=518), with most evidence derived from mild traumatic brain injury cohorts, and fewer studies involving stroke, mixed etiologies, and disorders of consciousness. Across domains, antisaccade and related paradigms were commonly associated with differences in inhibitory control and executive function, while predictive tracking, smooth pursuit, and target-blanking paradigms frequently captured alterations in attentional prediction and timing. Virtual reality (VR) free-viewing paradigms identified visuospatial exploration asymmetries in stroke-related neglect, and gaze-based human-computer interface approaches demonstrated above-chance task performance in a subset of patients with disorders of consciousness. Evidence for incremental validity beyond conventional assessment was mixed and often indirect, and safety reporting was uncommon. Overall certainty of evidence was generally low and limited by small sample sizes, cross-sectional designs, and heterogeneity in acquisition procedures, metrics, and analytic pipelines.

**Conclusions:**

Eye tracking shows potential as an adjunctive, process-level approach for quantifying specific cognition-relevant behaviors after ABI, particularly within paradigms targeting inhibitory control and predictive attention. Current evidence is insufficient to support broad diagnostic claims or the routine replacement of conventional neuropsychological assessment. Future research should prioritize harmonized paradigms and reporting standards, external validation of classification models, longitudinal designs, and explicit feasibility and safety reporting to clarify when eye tracking provides incremental clinical value for precision neurorehabilitation.

## Introduction

### Background

Acquired brain injury (ABI) is an important health concern, and its definition varies according to whether the medical condition is present at birth [1,2]. It includes all types of brain injury occurring after birth and not due to genetic or congenital pathology [3]. Unlike progressive neurological disorders such as Alzheimer disease or Parkinson disease, ABI is typically induced by an event that disrupts the normal function of the brain and results in changes to neuronal activity that can be observed at the physical, metabolic, or functional level of the nerve cells [1-3]. There are 2 main categories under ABI, which include traumatic brain injury (TBI) and nontraumatic brain injury (nTBI) [4]. Among the most common causes of TBI in older children and adults are assaults, falls, and crashes in motor vehicles, as well as sports injuries and blast injuries, including military blast injuries [5]. nTBI is an umbrella term that includes brain lesions seen in strokes in which blood enters but does not reach brain tissue; infections (eg, meningitis and encephalitis); hypoxia or anoxia, where the brain is deprived of oxygen; brain tumors; exposure to toxins; and drug or alcohol abuse [6]. Epidemiological data show that ABI is a global health problem and one of the major causes of morbidity and mortality [7,8]. More than 20 million new cases of TBI alone are estimated to occur each year worldwide; in fact, some estimates put the overall number of all TBIs, whatever their cause, at between 64 and 74 million annually [9]. TBI can be mild, moderate, or severe based on the score on the Glasgow Coma Scale or the duration of loss of consciousness or posttraumatic amnesia [10,11]. In addition to traditional clinical scales, motor and cognitive functional outcomes are significant limitations when trying to predict long-term recovery following ABI [12]. As many as 65% of individuals with moderate-to-severe TBI continue to experience chronic cognitive impairments, most likely to impact memory (20%‐79%), attention (18%‐54%), processing speed (17%‐19%), and executive functioning (eg, planning, problem solving, and multitasking) during the chronic phase of recovery [13]. In the motor domain, more than one-third of people with severe TBI still present with signs of neuromotor dysfunction (eg, paresis, ataxia, and postural abnormality) two years following brain injury [14,15]. These cognitive disturbances and motor deficits directly impact gait stability, fine motor coordination, and activities of daily living, underscoring the need for precise assessment tools [16].

Rehabilitation after ABI is increasingly characterized by technology-assisted interventions that aim to support cognitive and functional recovery. Recent syntheses highlight growing interest in noninvasive brain stimulation, virtual and extended reality–based rehabilitation, computerized cognitive training, and robot-assisted interventions, while also emphasizing heterogeneity in protocols, target domains, and patient profiles [17-20]. This evolving landscape strengthens the need for outcome measures that are sensitive to change, feasible across clinical settings, and capable of capturing real-time performance during task engagement.

Eye-tracking technology, characterized by excellent temporal and spatial resolution, not only allows for the sensitive detection of subtle oculomotor abnormalities associated with these functional deficits but also can be used as an assistive interface to restore communication for patients lacking volitional motor control [21]. Eye-tracking systems, which convert eye movements into control signals, can also assist with assessment, environmental control, communication, and rehabilitation exercises, enabling smooth transition between assessment and intervention and providing the connection to the applications [22]. Eye-tracking technology enables the measurement of various aspects of eye behavior (eg, eye gaze and pupil size) and has been applied in psychology research to study visual attention and cognitive processing states [23-25]. Often, the technology involves the use of infrared cameras and light sources that track the pupil and corneal reflections, which are then translated into data streams that allow for information about various cognitive states [26,27]. There are various types of eye trackers available, with options designed for controlled laboratory settings, real-world environments, virtual reality (VR) or augmented reality headsets, high-precision head-stabilized systems, and more accessible webcam-based trackers [28-31]. Eye tracking measures key oculomotor parameters such as fixations, saccades, pupillometry, smooth pursuit, vergence, blinks, and other eye movements (gaze paths) [32,33]. In cognitive assessment, eye tracking has been used to screen attention, memory, executive functions, language processing, and reading fluency and has demonstrated promise in the early diagnosis of neurodegenerative diseases [34,35]. Eye-tracking technology has particular implications for cognitive assessment in cases of ABI. Its usefulness has been extended to detect oculomotor dysfunction due to mild traumatic brain injury (mTBI), including concussive injuries [36]. It has also proven useful for determining levels of consciousness in patients with extreme brain injury, as well as for examining spatial biases in visual exploration after stroke [37].

### Rationale, Objective, and Theoretical Framework for Investigating Eye Tracking in ABI Cognitive Assessment

As ABI represents a substantial global health problem, the previous overview shows that eye-tracking technology can be a useful and powerful tool to explore cognitive states through oculomotor behavior. Conventional neuropsychological assessments remain central to cognitive evaluation after ABI. Nevertheless, administration and interpretation can be challenging in individuals with motor impairment, aphasia, reduced stamina, or fluctuating arousal, which may limit feasibility and measurement precision [34]. Sensitivity can also be reduced for subtle deficits that are common after mTBI, and repeated testing can be influenced by practice effects. Scoring and interpretation may vary across raters and clinical contexts, and many instruments provide only a snapshot rather than capturing dynamic cognitive control during continuous interaction with the environment. In contrast, eye tracking offers a potentially less demanding, systematic, and more objective approach to assess some cognitive functions, such as attention, memory, and executive control, through the direct measurement of neural processing correlates manifested in gaze patterns (fixation duration, saccadic patterns, and pupillary responses) [35]. However, although promising applications and research are emerging and interest in the field of ophthalmoplegia continues to grow, the available evidence supporting the implementation of eye tracking for cognitive assessment across the broad spectrum of ABI, including multiple etiologies (TBI and nTBI), levels of severity, and phases of recovery, remains mixed. Importantly, ABI encompasses heterogeneous etiologies and clinical phenotypes, including traumatic injury, stroke, hypoxic-ischemic injury, and disorders of consciousness (DoC), with meaningful variation by severity and phase of recovery. This heterogeneity can influence both the choice of eye-tracking paradigm and the interpretation of findings, underscoring the need to synthesize evidence with attention to ABI subgroup and target cognitive domain. There is a gap in the literature regarding a consolidated synthesis of this evidence. Therefore, this study aimed to identify and synthesize the available literature on the use of eye-tracking technology for cognitive assessment in populations with ABI. This work is based on a theoretical foundation that closely links oculomotor control to cognitive functioning, such that ABI-related alterations are thought to reflect measurable changes in eye movement patterns, which in turn are related to the affected cognitive domains [36,37]. From a neurobiological perspective, oculomotor behavior is governed by distributed control systems that overlap with cognitive networks. Antisaccade paradigms rely on frontostriatal circuitry and executive inhibition, whereas smooth pursuit and predictive tracking recruit cerebellar and parietal contributions that support timing, prediction, and attentional allocation. Fixation stability, gaze variability, and pupil dynamics can index distinct components of cognitive processing rather than a single unitary construct. These mechanistic links support the rationale for using eye tracking as a process-level window into cognition, provided that paradigms and metrics are interpreted within their specific neurocognitive targets [38,39]. This effort should allow for a thorough evaluation of the existing scientific evidence and help guide future researchers and clinicians in the rehabilitation process of this patient population. Table 1 provides an overview of eye-tracking technologies [40-48].

**Table 1.** Overview of eye-tracking technologies.

Eye tracking technology	Description	Mechanism of action	Relevance to ABI^a^ cognitive assessment	Advantages and limitations	Clinical applications
Tobii 5 (laptop-mounted) [40]	Binocular eye tracker (133 Hz^b^) with head tracking (33 Hz), mounted on a laptop.	Infrared tracking of pupil and corneal reflection; calibrated using Tobii Pro Lab software (Tobii AB).	Captures changes in gaze stability and saccade latency, useful post injury.	Portable, high sampling rate, and limitation may be sensitive to ambient lighting.	Used for fixation and saccadic movement assessment after ABI or mTBI^c^.
RightEye System [41]	Computerized eye-tracking system evaluating oculomotor behavior.	Digitally tracks smooth pursuits, saccades, vergence, and reaction times.	Assesses oculomotor deficits and symptom severity in RHI^d^ and concussion.	Comprehensive eye metrics, and limitation may be less sensitive to subtle impairments.	Concussion and neurovisual symptom assessment.
BEAM Task Eye Tracker [42]	Automated tracking system integrated with BEAM^e^ cognitive tasks.	Monitors saccades in response to visual cognitive tasks on screen.	Correlates with neuropsychological symptoms (eg, depression and PTSD^f^).	Continuous, task-integrated, and limitation may be task-dependent metrics.	Mental health–linked eye tracking in the ABI context.
Tobii T60/T60 [43]	Seventeen-inch monitor with noninvasive eye-tracking unit.	Infrared beams detect gaze location and fixations.	Detects fixation patterns and attention to scenes and grids.	Stable and accurate, and limitations may be fixed setup and screen size.	Visual attention and scene perception in ABI research.
Tobii EyeX [44]	Monitor-mounted tracker (>60 Hz) for fixation-point recording.	Infrared corneal reflection for eye-gaze coordinates.	Differentiates visual fixation across GCS^g^ levels.	Affordable, good for basic research, and the limitation may be lower resolution.	Fixation-based cognitive-status screening.
HTC Vive with Pupil Labs [45]	VR^h^ headset with integrated eye tracking for immersive testing.	IR^i^ eye cameras track gaze with 1° accuracy in virtual environments.	Assesses VR-based gaze symmetry, fixation, and orientation in patients.	Ecological validity, and the limitation may be potential motion sickness and setup cost.	VR cognitive assessment and rehabilitation for ABI/mTBI.
EyeLink 1000 [46]	High-speed eye tracker (up to 1000 Hz) and head-stabilized.	IR beam reflection records eye movements with high temporal precision.	Tracks reaction times and fixation accuracy in cognitive tasks.	High precision and limitation may require head stabilization.	Detailed oculomotor assessment in laboratory settings.
Gazepoint GP3 HD [47]	150 Hz eye tracker with 9-point calibration screen.	Measures saccades and working memory through gaze behavior.	Differentiates performance in working memory and inhibition tasks.	Compact and high frequency, and limitation may miss microsaccades.	Working memory and executive function testing in ABI.
Applied Science Laboratories D6 [48]	High-speed desktop tracker for effort testing.	Records gaze patterns to detect malingering/fake effort.	Validates effort and symptom exaggeration in mTBI.	Reliable classification and limitation may be specialized purpose.	Effort testing and malingering detection.

a ABI: acquired brain injury.

b HZ: Hertz.

c mTBI: mild traumatic brain injury.

d RHI: right hemisphere infarction.

e BEAM: Bethesda Eye and Attention Measure.

f PTSD: posttraumatic stress disorder.

g GCS: Glasgow Coma Scale.

h VR: virtual reality.

i IR: infrared.

## Methods

### Protocol and Reporting Standard

This systematic review was conducted and reported in line with PRISMA (Preferred Reporting Items for Systematic Reviews and Meta-Analyses) 2020 guidance, with all stages planned to maximize transparency and reproducibility [49]. The review protocol was prospectively registered in PROSPERO (CRD420251038768) to reduce the risk of selective reporting and to provide an auditable record of the prespecified methods [50]. Any deviations from the registered protocol were documented and justified within the paper to preserve methodological transparency [49,50]. Following peer-review feedback, the PROSPERO record was updated on February 26, 2026, to clarify eligibility criteria, including comparator requirements and inclusion of eligible case series, and to specify the design-appropriate risk-of-bias tools used in this review.

### Review Question Framework (PICO)

To ensure conceptual clarity and consistent eligibility decisions, we framed the review question using a PICO-informed structure adapted for diagnostic and assessment technologies. The population comprised children and adults with ABI across etiologies, severities, and recovery phases. The index assessment modality was eye tracking, used as the primary approach to quantify cognition or clinically relevant cognitive-communication processes, with paradigms and derived metrics prespecified and mapped to cognitive domains. Comparators were not mandatory and, when available, included healthy controls, ABI subgroups, within-participant conditions (eg, baseline vs follow-up), and/or conventional neuropsychological measures. Outcomes were eye-tracking–derived metrics relevant to cognition (eg, antisaccade latency or error rates for inhibitory control, predictive tracking measures for attentional timing, and fixation or exploration indices for visuospatial processing), along with diagnostic or classification performance indices when explicitly reported. This framework guided study selection, data extraction, and synthesis and reduced the risk of misclassifying eye tracking as an intervention rather than an assessment approach.

### Eligibility Criteria and Operational Definitions

We included peer-reviewed, full-text studies published in English that enrolled children or adults with ABI of any etiology, severity, and recovery phase. Eligible etiologies encompassed TBI, stroke, hypoxic-ischemic injury, infectious or inflammatory ABI, and DoC following ABI. Studies were eligible when eye tracking was used as the primary assessment modality to quantify cognitive functioning. Eye tracking was operationally considered “primary” when the paradigm and derived oculomotor metrics were central to the cognitive assessment objective and were used to operationalize at least one cognitive domain or a clinically meaningful cognitive-communication construct. For the purposes of this review, eligible cognitive targets included attention, executive function, memory and working memory, visuospatial exploration and neglect-related processes, language and cognitive-communication processes, social-cognitive paradigms, and gaze-based paradigms aimed at detecting command following, volitional control, or awareness in DoC. We included randomized controlled trials, nonrandomized intervention studies, and observational designs, including cohort, cross-sectional, and case-control studies.

We excluded protocols, conference abstracts, dissertations, reviews, animal studies, and single-case reports. We also excluded studies in which eye tracking was used only for technical device validation, usability testing without cognitive interpretation, or oculomotor characterization without an explicit link to a cognitive target or classification framework. Studies that included mixed neurological samples were eligible only when ABI data were reported separately or could be unambiguously extracted.

### Information Sources and Search Strategy

We searched PubMed, Web of Science, the Cochrane Library, Embase, EBSCOhost, PsycINFO, and Scopus from database inception to April 10, 2025. The initial search was performed on March 5, 2025 and updated on April 10, 2025. No publication date restrictions were applied. Searches combined controlled vocabulary terms and free-text keywords related to eye tracking, oculomotor metrics, cognition, and cognitive assessment, and ABI and related etiologies. The strategy was iteratively refined to optimize sensitivity while preserving relevance, and the complete search strategy is reported in Multimedia Appendix 1.

To reduce the risk of missing relevant studies, the reference lists of included studies and of closely related reviews were screened, and forward citation tracking was performed for key sentinel studies identified during full-text screening. Only peer-reviewed full-text papers were included to align with the prespecified scope and to ensure interpretability of methods and results.

### Study Selection

Records were exported to a single dataset and deduplicated using multiple identifiers, including title, authorship, year, and digital object identifier when available. Two reviewers (AC and RDL) independently screened titles and abstracts, followed by full-text assessment against eligibility criteria. Before formal screening, a calibration phase was performed to ensure consistent application of criteria. Disagreements were resolved through discussion, with adjudication by a third reviewer (RSC) when needed. Interrater agreement at the abstract and full-text stages was quantified using the Cohen kappa statistic, and agreement was interpreted using established benchmarks to support transparent reporting of selection reliability [51,52]. Reasons for exclusion at the full-text stage were recorded and are available upon request.

### Data Extraction and Data Items

Two reviewers (AC and RDL) independently extracted data using a piloted extraction form, with discrepancies resolved by consensus and third-reviewer adjudication (RSC) when necessary. Extracted study-level information included design, setting, recruitment procedures, sample size, participant demographics, ABI etiology, severity indices where reported, and phase of recovery at assessment.

Eye-tracking–specific data were extracted at a granular level to support clinical interpretability and reproducibility. We recorded the eye-tracking platform and device model, sampling rate, calibration procedures, and success criteria when reported, head stabilization conditions, and the testing environment. We extracted paradigm characteristics, including task instructions, stimulus type, trial structure and duration, and any concurrent task demands such as dual tasking or working memory load manipulations. We extracted primary and secondary oculomotor metrics and documented how each metric was operationalized by the original authors. We then mapped each paradigm and metric to the prespecified cognitive target domain using the authors’ stated rationale and the task requirements.

Comparator conditions were recorded when present, including healthy controls, ABI subgroups, baseline within-participant comparators, and conventional neuropsychological instruments used as reference measures. We extracted statistical information required for synthesis, including group differences, within-subject changes, correlation coefficients, regression coefficients, and classification performance metrics such as sensitivity, specificity, and area under the curve when reported. Feasibility and data quality indicators were extracted when available, including calibration failures, exclusion due to inadequate tracking quality, missing data rates, and any preprocessing or artifact handling described by the authors. Safety information was extracted and coded as not reported, explicitly absent, or reported with no adverse events to avoid misclassification of missing safety reporting as evidence of safety.

### Risk of Bias Assessment

Risk of bias was assessed independently by 2 reviewers using design-appropriate tools, with disagreements resolved by consensus and third-reviewer adjudication when needed. For randomized controlled trials, we applied Risk of Bias 2 across the standard domains and derived an overall judgment to inform interpretation of intervention effects [53]. For nonrandomized intervention studies, we applied Risk of Bias in Non-Randomized Studies of Interventions, with particular attention to confounding, selection bias, and outcome measurement, which are especially relevant in heterogeneous ABI populations [54].

For studies whose primary purpose was classification or diagnostic discrimination using eye-tracking metrics, we evaluated risk of bias and applicability using Quality Assessment of Diagnostic Accuracy Studies 2 (QUADAS-2), which is specifically structured to address concerns in patient selection, index test conduct and interpretation, reference standards, and flow and timing [55]. For observational cohort and case-control studies that did not meet diagnostic-accuracy criteria, we assessed methodological quality using the Newcastle-Ottawa Scale (NOS) to capture selection, comparability, and outcome or exposure ascertainment features that may drive systematic bias [56]. Risk-of-bias assessments were not used as exclusion criteria, but they were incorporated into synthesis and certainty judgments to maintain transparency in interpretation.

### Data Synthesis and Effect Measures

Given the expected heterogeneity in paradigms, metrics, and ABI subgroups, we prespecified a primarily narrative synthesis structured by cognitive domain and eye-tracking paradigm, with additional stratification by etiology and recovery phase where feasible. Narrative synthesis followed established principles for integrating quantitative and qualitative evidence, with emphasis on consistency of direction and robustness across designs rather than vote counting [57].

When quantitative pooling was feasible, we planned meta-analysis only when at least 3 studies used sufficiently comparable paradigms, reported the same outcome metric, and provided adequate summary data. For continuous outcomes, we planned to compute standardized mean differences for between-group comparisons or standardized mean change for within-subject designs, with 95% CIs. Random-effects models were prespecified, given expected clinical and methodological diversity. When meta-analysis was not feasible, we summarized findings descriptively and reported effect sizes as presented by the original studies, clarifying the metric and its interpretation. Diagnostic performance indices such as sensitivity, specificity, and area under the curve were summarized descriptively and were not pooled due to heterogeneity in thresholds, paradigms, and reference standards.

### Certainty of Evidence

Certainty of evidence was evaluated using GRADE principles, considering risk of bias, inconsistency, indirectness, imprecision, and publication bias [58,59]. Certainty ratings were planned for each main outcome group aligned with the synthesis structure, with judgments explicitly justified and linked to the corresponding risk-of-bias assessments. Ratings were planned to be presented in a summary of findings table to support transparent translation of the evidence base into clinical interpretability [58,59]. The methodological framework used in this systematic review is summarized in Table 2.

**Table 2.** Detailed summary of the systematic review methodology.

Section methodology	Details
Protocol and reporting	Reporting standard: PRISMA^a^ 2020 [49]. Registration: prospective registration in PROSPERO^b^ (CRD420251038768) [50].
Inclusion criteria	Participants: children and adults with acquired brain injury (ABI) of any etiology, severity, and recovery phase, including traumatic brain injury, stroke, hypoxic-ischemic injury, and disorders of consciousness after ABI. Technology: studies in which eye tracking was used as the primary assessment modality to quantify cognitive functioning. Operational definition: eye tracking was considered primary when the paradigm and derived oculomotor metrics were central to the cognitive assessment objective and were used to operationalize at least one cognitive domain or a clinically relevant cognitive-communication process. Cognitive targets: attention, executive function, memory and working memory, visuospatial exploration and neglect-related processes, language and cognitive-communication processes, social-cognitive paradigms, and gaze-based paradigms aimed at detecting command following, volitional control, or awareness in disorders of consciousness. Eye-tracking metrics: studies reporting defined oculomotor outcomes (eg, fixations, saccades, smooth pursuit, vergence, or pupillary dynamics) with an explicit cognitive interpretation, including associations with conventional cognitive measures and/or discrimination or classification performance. Study designs: randomized controlled trials, nonrandomized intervention studies, and observational designs (cohort, cross-sectional, and case-control). Language: full-text papers published in English.
Exclusion criteria	Focus of study: studies not centered on cognitive assessment using eye tracking in ABI populations. Population: nonhuman studies, non-ABI samples, or mixed neurological samples without extractable ABI-specific data. Technology use: studies using eye tracking only for technical device validation or usability testing without a cognitive framework, or studies describing oculomotor dysfunction without an explicit link to a cognitive target or classification approach. Publication type: protocols, conference abstracts, dissertations, reviews, and single-case reports or case series.
Review question framework	Population: individuals with ABI across etiologies, severity levels, and recovery phases, including pediatric and adult samples. Index assessment: eye-tracking paradigms and derived oculomotor metrics used to quantify cognitive domains or cognitive-communication constructs. Comparator or reference: healthy controls, ABI subgroups, within-participant baselines, and/or conventional neuropsychological measures when available. Outcomes: domain-relevant cognitive inferences from eye-tracking metrics, associations with conventional assessments, group discrimination or classification performance (eg, sensitivity, specificity, and area under the curve), and feasibility and safety indicators.
Search period	Coverage: database inception to April 10, 2025, with no publication date restrictions. Initial search: March 5, 2025. Update search: April 10, 2025. Information sources: PubMed, Web of Science, the Cochrane Library, Embase, EBSCOhost, PsycINFO, and Scopus. Full database-specific strategies are reported in Multimedia Appendix 1.
Study selection	Deduplication: records were collated and deduplicated before screening. Screening: 2 reviewers independently screened titles and abstracts, followed by full-text eligibility assessment, with third-reviewer adjudication when needed. A PRISMA 2020 flow diagram documented the selection process [49]. Reliability: interrater agreement was quantified using the Cohen kappa statistic and interpreted using established benchmarks [51,52].
Data extraction	Process: 2 reviewers independently extracted data using a piloted extraction form, with discrepancies resolved by consensus and third-reviewer adjudication when required. Study-level items: design, setting, sample size, participant demographics, ABI etiology, severity indices where reported, and phase of recovery. Eye-tracking technical items: device model or platform, sampling rate, calibration procedure, and success criteria when reported, head stabilization conditions, and testing environment. Paradigm and outcomes: task procedures and stimulus characteristics, trial structure and cognitive load, primary eye-movement metrics, and mapping of paradigms and metrics to cognitive targets. Comparators and statistics: comparator conditions (eg, controls, ABI subgroups, within-participant baselines, and conventional tests) and extractable statistics (group differences, within-subject changes, correlations, regression coefficients, and diagnostic performance indices when reported).
Risk of bias assessment	Randomized trials: RoB 2^c^ [53]. Nonrandomized interventions: ROBINS-I^d^ [54]. Diagnostic accuracy or classification studies: QUADAS-2^e^ [55]. Observational cohort or case-control studies: Newcastle-Ottawa Scale [56]. Process: 2 reviewers assessed risk of bias independently, with disagreements resolved by consensus and third-reviewer adjudication when necessary.
Data synthesis	Primary approach: structured narrative synthesis organized by cognitive domain and eye-tracking paradigm, with stratification by etiology and recovery phase where feasible, following established narrative synthesis principles [57]. Quantitative synthesis: meta-analysis was planned only when at least 3 studies used sufficiently comparable paradigms and reported the same outcome metric with adequate summary data; random-effects models were prespecified for continuous outcomes. Diagnostic indices: classification and diagnostic performance metrics were summarized descriptively and were not pooled due to heterogeneity in thresholds, paradigms, and reference standards.
Safety and feasibility	Safety: adverse events were extracted and coded as not reported, explicitly absent, or reported with no adverse events. Feasibility: calibration failures, tracking-quality exclusions, and missing data were extracted when reported.
Certainty of evidence	Approach: certainty of evidence for each main outcome group was evaluated using GRADE^f^ principles [58,59]. Reporting: certainty ratings were planned to be presented in a summary of findings table.

a PRISMA: Preferred Reporting Items for Systematic Reviews and Meta-Analyses.

b PROSPERO: International Prospective Register of Systematic Reviews.

c RoB 2: Risk of Bias 2.

d ROBINS-I: Risk of Bias in Non-Randomized Studies of Interventions.

e QUADAS-2: Quality Assessment of Diagnostic Accuracy Studies 2.

f GRADE: Grading of Recommendations, Assessment, Development, and Evaluations.

### Ethical Considerations

As this systematic review involved secondary data analysis from previously published studies, no new ethical approval was required.

## Results

### Study Selection

**Figure 1.**
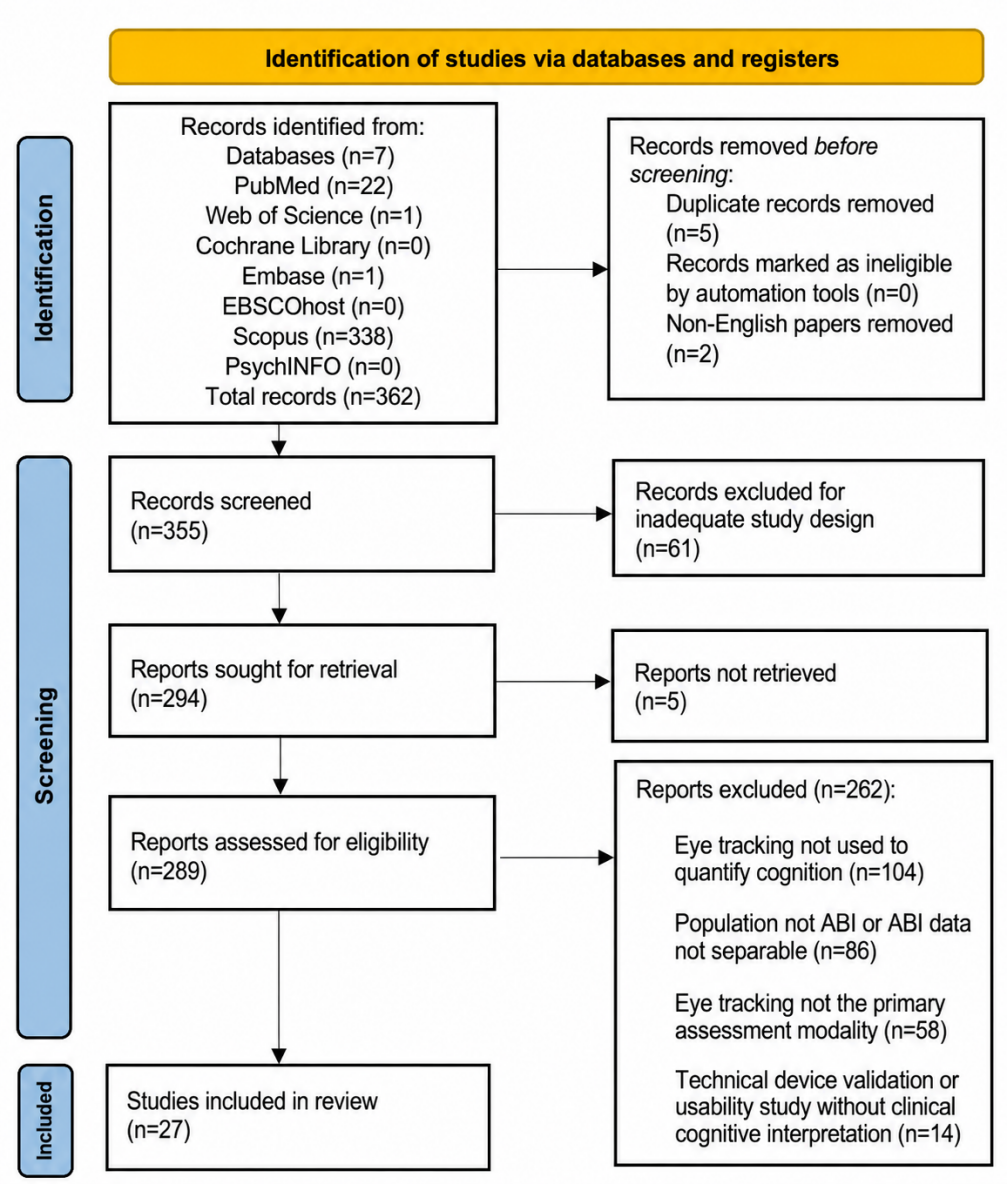
PRISMA (Preferred Reporting Items for Systematic Reviews and Meta-Analyses) 2020 flow diagram of evaluated studies. ABI: acquired brain injury.

The electronic search across 7 databases identified 362 records. After removal of 5 duplicates and 2 non-English records, 355 records underwent title and abstract screening. At this stage, 61 records were excluded due to inadequate study design, leaving 294 reports sought for retrieval. Despite targeted retrieval efforts, 5 reports could not be obtained, and 289 full-text reports were assessed for eligibility. Of these, 262 reports were excluded for prespecified reasons, most commonly because eye tracking was not used to quantify cognition (104 reports), the population was not ABI or ABI-specific data were not separable (86 reports), eye tracking was not the primary assessment modality (58 reports), or the paper focused on technical and device validation or usability without clinical-cognitive interpretation (14 reports). This process yielded 27 studies that met eligibility criteria and were included in the qualitative synthesis [60-86]. The study selection process is summarized in Figure 1.

### Characteristics of Included Studies and Participants

The included evidence base (refer to Multimedia Appendix 1) was heterogeneous in both clinical populations and methodological approaches, with a strong predominance of TBI cohorts and fewer studies in nontraumatic ABI subgroups. Across included studies, a total of 872 participants were enrolled, including 354 females and 518 males, although sex was not consistently reported or analyzed as an effect modifier across all cohorts [60-86]. Most studies focused on mTBI, concussion, or persistent postconcussive symptoms, often in military or athletic samples [60-62,68-75,78-83,85]. Moderate-to-severe TBI was represented in a smaller subset of studies, generally using case-control paradigms to examine cognitive-communication or higher-order integration processes [63,64,67,76,77]. Stroke was represented primarily through a single visuospatial neglect study using a VR free-viewing paradigm [66], while DoC were represented through gaze-based paradigms and human-computer interface (HCI) approaches designed to probe command following or awareness in patients with severe impairments [65,84]. One rehabilitation study enrolled adults with mixed etiologies (TBI and stroke) and examined oculomotor rehabilitation for reading-related deficits [86]. A single pediatric study examined mild closed head injury and tracked saccadic outcomes and cognitive performance longitudinally for more than 6 months [80]. Pediatric evidence was limited to one longitudinal study in children with mild closed head injury, which assessed saccadic performance and memory outcomes at acute, 3-month, and 6-month follow-up. This study suggested that developmental factors and age subgroup effects may influence oculomotor trajectories, with a pattern consistent with altered maturation or practice effects rather than a uniform deficit profile across all pediatric participants [80]. Because pediatric data were sparse and paradigms differed from those used in adult cohorts, findings in children should be interpreted cautiously and not generalized to the broader ABI spectrum without additional age-stratified studies using harmonized paradigms and reporting.

Study sizes were typically small to moderate. Median sample size across studies was 45 participants (range 10‐188), with 15 of 27 studies enrolling fewer than 50 participants and only 2 studies enrolling 100 or more participants [62,85]. Most studies were cross-sectional, with fewer longitudinal designs or repeated-measures components. Longitudinal or baseline-to-follow-up approaches were used in concussion cohorts to relate within-individual changes in eye-tracking metrics to symptom burden [79,83], and one pediatric study implemented repeated assessments at acute, 3-month, and 6-month time points [80]. The evidence base, therefore, offers breadth in paradigms and outcomes, but it remains uneven across etiologies, severity strata, and recovery phases, with limited prospective follow-up outside concussion-focused cohorts.

### Results by Cognitive Domain and Clinical Use Case

Given the heterogeneity of paradigms and outcome definitions, no quantitative pooling was performed. Findings are therefore summarized narratively and organized by cognitive domain and clinical application, with attention to consistency across paradigms, ABI subgroups, and the extent to which eye-tracking outcomes were evaluated alongside conventional assessments.

### Executive Function and Inhibitory Control

Evidence relevant to executive function and inhibitory control was derived largely from antisaccade and related paradigms, as well as from task batteries embedding oculomotor indices under attentional demands [68,73-77,80,83]. Across adult mTBI and chronic TBI samples, antisaccade paradigms generally demonstrated increased latency and/or elevated error rates relative to controls, consistent with impaired inhibitory control and executive regulation [74,76,83]. In chronic cohorts, executive dysfunction was often most apparent under task conditions requiring suppression of reflexive saccades or sustained inhibitory control, with several studies indicating that oculomotor indices captured impairments not always mirrored by global cognitive screening measures. In one exploratory study integrating antisaccade performance with diffusion measures and Stroop interference, longer antisaccade latencies were associated with greater symptom burden and poorer interference control in acute mTBI and were strongly correlated with white matter integrity markers in the splenium of the corpus callosum within the acute subgroup [83]. These observations support the plausibility that oculomotor inhibition metrics can reflect executive control processes and their neurobiological correlates in at least a subset of mTBI presentations.

Age- and severity-related modulation emerged as important contextual factors. In one study using embedded saccadic measures, older age was associated with slower saccadic response times and reduced inhibitory performance among those with a history of mTBI, suggesting that eye movement–based indices may capture vulnerability patterns that interact with age-related changes in attentional control [75]. Severity-dependent effects were also reported in chronic TBI cohorts, with moderate-to-severe groups typically exhibiting broader impairment profiles than mild TBI groups under similar saccade paradigms [76,77]. These findings are consistent with an injury-severity gradient, but they also highlight that the magnitude and detectability of inhibitory deficits depend on task selection, recording parameters, and analytic definitions.

### Working Memory and Cognitive Load Effects

A smaller evidence base evaluated working memory or cognitive load effects through paradigms explicitly manipulating attentional demand, most commonly using n-back designs with embedded oculomotor outcomes [68,73] and related load-dependent paradigms [72]. Across these studies, oculomotor impairment tended to become more apparent as cognitive load increased. In chronic TBI cohorts, saccadic latency and error indices worsened at higher load levels, with greater impairment observed in more severe injury groups and with findings suggesting that load manipulations increase the sensitivity of eye movement indices to cognitive vulnerability [73]. A clinically oriented multimodal assessment incorporating a cognitively demanding paradigm reported that specific saccadic indices contributed to discrimination between chronic mTBI and controls, with conventional neuropsychological measures showing less consistent separation in that setting [68]. Evidence in this domain remains limited by the small number of studies and by task-specific implementations, but the direction of findings is broadly coherent with the notion that cognitive load amplifies executive and attentional demands reflected in oculomotor control.

### Attention and Predictive Control

The largest body of evidence relevant to attention, predictive control, and attentional timing involved smooth pursuit, predictive visual tracking, target blanking, and gap paradigms designed to probe anticipatory control [70,72,74,78,79,81,82]. Several studies reported that mTBI or postconcussive symptom groups demonstrated altered pursuit or visual tracking performance, particularly under conditions that required prediction of target trajectories or compensation for missing visual input. In 2 studies using predictable smooth pursuit paradigms, mild TBI groups showed reduced target prediction and increased eye position error and variability relative to controls, with correlations between pursuit-related indices and verbal learning measures related to attention and executive functioning [81,82]. In a predictive tracking task coupled with magnetoencephalography, deficits were most pronounced under gap conditions, suggesting that anticipatory control may be selectively impaired when the paradigm reduces reliance on continuous sensory feedback and increases top-down prediction demands [72]. Similarly, a visual tracking paradigm used in concussion monitoring showed that within-individual changes in tracking metrics were moderately to strongly correlated with symptom burden when assessed within 2 weeks post injury, whereas simple reaction time metrics showed weaker relationships to symptom scales despite measurable post injury slowing in throughput measures [79]. Together, these findings suggest that predictive timing and sustained attention components of pursuit or tracking paradigms may map more closely to symptom profiles than simple reaction-based outcomes in some concussion contexts.

Not all evidence converged on robust group differences. One study combining diffusion tensor imaging, neurocognitive assessment, and visual tracking reported that the concussion cohort showed largely comparable tracking performance to controls, despite imaging differences and evidence of slowed attention-related reaction time, indicating that tracking metrics may be sensitive in some cohorts but not uniformly abnormal across mTBI presentations [78]. In another study, behavioral eye measures were less discriminatory than imaging-derived indices in separating chronic mTBI from controls, underscoring that the comparative performance of eye tracking depends on the specific paradigm and the selected outcome metric [70]. These mixed findings collectively support the need to interpret attentional tracking outcomes as paradigm- and population-dependent rather than as uniform markers of impairment.

### Visuospatial Exploration and Neglect Detection

Evidence for visuospatial exploration was limited but clinically instructive. In a VR free-viewing museum environment, stroke patients with spatial neglect demonstrated lateralized gaze and head-orientation asymmetries relative to controls, and the approach was described as capable of detecting atypical exploration patterns that were not captured consistently by conventional tests in all individuals [66]. This study supports the feasibility and potential incremental utility of immersive, naturalistic paradigms for detecting spatial biases, although generalizability remains limited because the evidence in this domain is currently based on a single study and a single implementation approach.

### Cognitive-Communication and Social Cognition

Three studies examined cognitive-communication or socially relevant visual attention patterns through tasks that were not reducible to classic executive function paradigms, but nevertheless quantified cognition through structured gaze behavior [63,64,67]. In a visual-world paradigm assessing online speech–gesture integration, both groups showed facilitation from meaningful gestures, but this effect was attenuated in moderate-to-severe TBI, suggesting reduced integration of multimodal cues during language processing [67]. In severe TBI cohorts evaluating visual attention during interpretation of augmentative and alternative communication displays, grid-based formats were associated with greater visual effort than scene-based formats in both groups, and TBI participants showed less efficient gaze patterns overall, consistent with increased processing demands and reduced efficiency in extracting relevant information [63]. A separate free-viewing study focusing on engagement cues in contextual photographs showed that both groups oriented rapidly to human figures and adjusted fixation patterns according to task-relevant engagement content, with limited between-group differences, suggesting that some aspects of social attention may be relatively preserved depending on task demands and stimulus properties [64]. Taken together, this evidence indicates that eye tracking can quantify differences in processing efficiency and cue integration in cognitive-communication contexts, but the limited number of studies and the task-specific nature of outcomes constrain broad conclusions.

### DoC and Gaze-Based Paradigms for Command Following

Two studies focused on DoC populations and used gaze-based paradigms to probe command following or awareness in contexts where conventional assessment is challenged by limited motor output [65,84]. In a gaze-tracking HCI approach designed for DoC, a subset of patients performed above chance on one or more structured tasks, and the authors highlighted the potential for such tools to complement bedside diagnosis and reduce misclassification, including identification of patients who might retain meaningful command-following capacity [84]. A second study combined gaze-tracking tasks with complementary neurophysiologic measures and suggested that multimodal features could relate to clinical status classifications in severe ABI populations [65]. The evidence in this domain remains limited by small samples, variable arousal, and setting-specific feasibility constraints, but the convergence of both studies on above-chance task completion in a subset of patients supports continued evaluation of eye-tracking HCI approaches as adjunctive assessment tools in severe disorders.

### Incremental Validity Relative to Conventional Assessment

Direct evidence of incremental validity beyond conventional neuropsychological testing was limited because many studies did not prespecify incremental validity as a primary analytic goal and because neuropsychological comparators varied across cohorts and settings. When conventional measures were included, they were often used as correlates rather than as formal reference standards. Still, several patterns emerged. First, in paradigms tightly linked to attentional prediction and executive inhibition, oculomotor indices often showed associations with symptom burden and executive test performance, suggesting that they can capture clinically relevant variance in domains that are difficult to quantify with brief assessments alone [72,79,83]. Second, some multimodal or embedded-task approaches reported that oculomotor indices contributed meaningfully to group discrimination or classification in settings where conventional cognitive batteries showed limited differentiation, although this was not uniform across platforms and paradigms [68,70]. Third, other studies indicated that symptom-based or vestibular-ocular screening measures may outperform certain computerized eye-tracking platforms for specific operational goals, emphasizing that incremental value is likely to be context dependent and may vary by hardware, paradigm, and target construct [61]. Collectively, the evidence supports cautious interpretation: eye tracking may add value for specific paradigms and populations, but the current literature does not justify a generalized claim that eye tracking routinely exceeds conventional testing across ABI.

### Safety, Feasibility, and Data Quality Reporting

Safety reporting was sparse across the included studies. Only 1 of 27 (4%) studies explicitly reported no harms, while 26 of 27 (96%) studies did not report adverse events in a way that allowed clear classification [60-86]. Feasibility reporting was more common but remained incomplete. Thirteen of 27 (48%) studies provided at least some feasibility or exclusion information related to calibration, equipment failures, incomplete testing, or analytic inclusion, whereas 14 of 27 (52%) studies did not report feasibility indicators beyond general study completion statements. Where reported, feasibility challenges included calibration failure and fatigue limiting completion in a VR neglect assessment [66], equipment-related exclusions in a language-processing study [67], incomplete administration of conventional tests due to physical impairment in a severe TBI cohort [63], and differential analytic sample sizes attributable to unusable eye-tracking records in a prospective concussion cohort [79]. Several paradigms required head stabilization, specialized equipment, or controlled testing conditions, which may limit scalability in routine clinical environments [67,70,74,81,82]. These findings underline that feasibility and access considerations are integral to interpretation and translation, and that standardized reporting of calibration success, data loss, and exclusions remains an important gap.

### Methodological Quality and Risk of Bias

**Figure 2.**
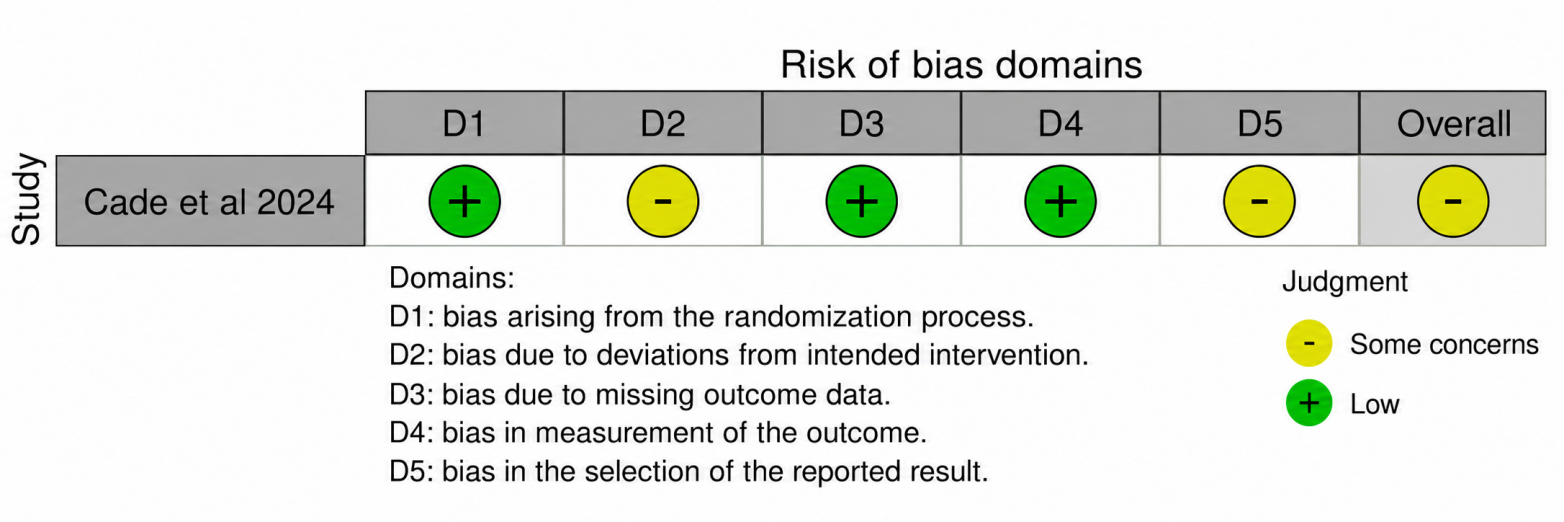
Risk of Bias of included randomized controlled trial studies [60].

Risk-of-bias assessments varied by study design and were interpreted with attention to the appropriate tool for each design. The complete risk-of-bias and methodological quality assessments are provided in Multimedia Appendix 2. The single randomized controlled trial was judged as having some concerns overall, reflecting limitations primarily related to deviations from intended interventions and the transparency of prespecified outcome reporting, while retaining low-risk judgments in several other domains of trial conduct and outcome measurement. Risk of Bias 2 domain-level judgments are provided in Figure 2.

Diagnostic and classification studies showed more substantial and heterogeneous concerns. QUADAS-2 assessments, summarized in Table 3, indicated a high risk of bias across all 3 classification studies, primarily driven by nonconsecutive or volunteer sampling strategies and by data-driven selection of predictors and decision thresholds. Applicability concerns were great for Barry and Ettenhofer [71] and Samadani et al [85] because the target condition and/or reference standards were not directly aligned with cognitive-status classification in ABI (performance-validity simulation in the former and computed tomography–defined injury status in the latter). Ettenhofer et al [68] showed lower applicability concerns because chronic mTBI status was established using a standardized identification approach with medical record confirmation, but risk of bias remained high due to in-sample variable selection procedures and exclusions that may inflate classification performance. These considerations suggest that reported classification estimates should be interpreted cautiously, particularly when models and thresholds are derived and evaluated within the same dataset, and external validation is not available [68,71,85].

**Table 3.** Quality Assessment of Diagnostic Accuracy Studies 2 (QUADAS-2) risk of bias and applicability assessment for diagnostic or classification studies. Judgments follow QUADAS-2 domains. Applicability concerns reflect relevance to the review question (eye-tracking metrics used for cognitive assessment or classification in acquired brain injury).

Study	Patient selection (RoB^a^ or applicability)	Index test (RoB or applicability)	Reference standard (RoB or applicability)	Flow and timing (RoB)	Overall (RoB)
Barry and Ettenhofer (2016) [71]	High or High	High or High	High or High	Low	High
Ettenhofer et al (2020) [68]	High or High	High or Low	Low or Low	High	High
Samadani et al (2015) [85]	High or Low	High or High	Low or High	Low	High

a RoB: risk of bias.

**Figure 3.**
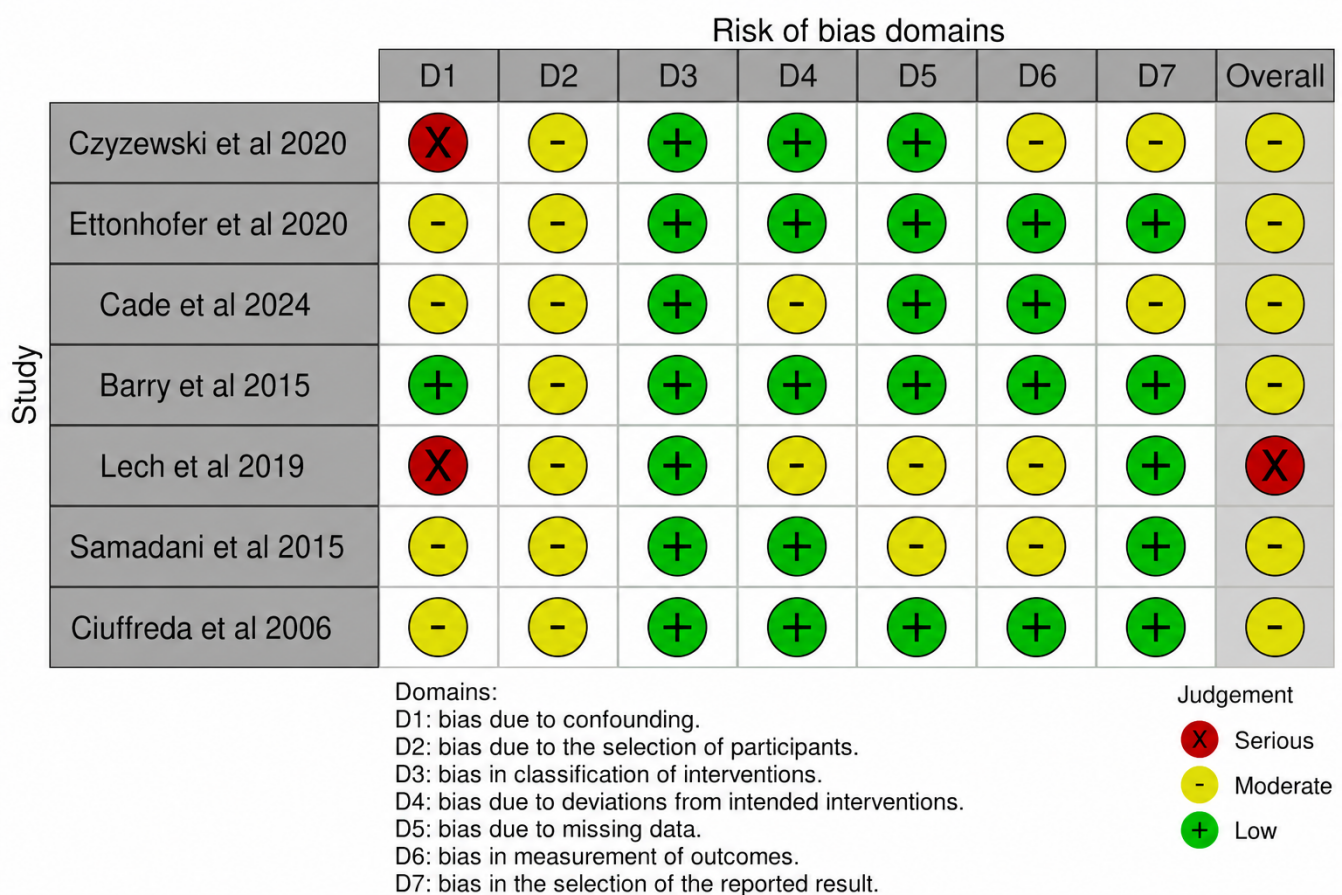
Cochrane Risk of Bias in Non-Randomized Studies of Interventions (ROBINS-I) [65,68,69,84-86].

Observational studies assessed with the NOS generally demonstrated fair methodological quality, with fewer studies achieving good ratings and 1 study rated as poor. Specifically, among the 19 observational studies assessed with NOS, 6 were rated good, 12 were rated fair, and 1 was rated poor, indicating recurring limitations in comparability and exposure or outcome ascertainment despite generally acceptable selection domains in many case-control designs [63,64,66,67,70,72-74,76-83]. NOS ratings are summarized in Table 4. Nonrandomized intervention studies [65,68,69,84-86] were assessed separately for risk of bias using an intervention-focused tool, and the domain-level judgments are provided in Figure 3. Across these designs, confounding and participant selection were common sources of concern, consistent with the predominance of small samples and heterogeneous inclusion criteria.

**Table 4.** Newcastle-Ottawa Scale (NOS) assessment for observational studies. NOS star ratings were applied in an adapted manner to accommodate nonrandomized observational designs commonly used in eye-tracking research. Overall quality labels are based on total stars (good: 7‐9, fair: 5‐6, and poor: <5).

Study	Design	Selection (0‐4)	Comparability (0‐2)	Exposure/outcome (0‐3)	Total (0‐9)	Overall quality
Astafiev et al (2015) [70]	Case-control	3	2	2	7	Good
Brown et al (2019) [63]	Case-control	3	2	2	7	Good
Clough et al (2024) [67]	Case-control	3	1	2	6	Fair
Diwakar et al (2015) [72]	Case-control	3	1	2	6	Fair
Ettenhofer et al (2018) [73]	Case-control	3	1	2	6	Fair
Heitger et al (2009) [74]	Case-control	3	1	2	6	Fair
Hershaw et al (2017) [75]	Case-control	3	1	2	6	Fair
Hougaard et al (2021) [66]	Case-control	4	1	2	7	Good
Hungerford et al (2023) [62]	Cross-sectional	3	2	2	7	Good
Kontos et al (2024) [61]	Case-control	3	1	2	6	Fair
Kraus et al (2007) [76]	Case-control	3	1	2	6	Fair
Kraus et al (2010) [77]	Case-control	3	1	2	6	Fair
Maruta et al (2016) [78]	Case-control	3	1	2	6	Fair
Maruta et al (2018) [79]	Cohort (single group)	2	0	2	4	Poor
Phillipou et al (2014) [80]	Case-control (longitudinal)	3	1	2	6	Fair
Suh et al (2006) [81]	Case-control	3	2	2	7	Good
Suh et al (2006) [82]	Case-control	3	2	2	7	Good
Thiessen et al (2017) [64]	Case-control	3	1	2	6	Fair
Ting et al (2016) [83]	Case-control	3	1	2	6	Fair

Certainty of evidence across outcome domains was generally low, driven by the predominance of nonrandomized designs, small samples, heterogeneity of paradigms and metrics, and limited external validation for classification analyses. Evidence was judged to be very low for most cognitive domains, with low certainty reserved for diagnostic or classification performance outcomes where classification metrics were reported but where risk-of-bias concerns and inconsistency limited confidence. Certainty ratings and primary reasons for downgrading are summarized in Table 5.

**Table 5.** Grading of Recommendations Assessment, Development and Evaluation (GRADE) certainty of evidence summary by cognitive domain and clinical use case. Certainty ratings reflect the overall body of evidence for each domain or use case. No quantitative pooling was undertaken; therefore, the summary reflects the direction and consistency of findings reported across studies. Domains may overlap because individual studies often reported multiple paradigms or outcomes.

Outcome domain or clinical use case	Evidence base (design and studies)	Summary of findings (narrative)	Certainty of evidence (GRADE^a^)	Main reasons for downgrading
Executive function and inhibitory control (eg, antisaccade performance, inhibition errors, and reaction time variability)	Primarily observational (case-control or cross-sectional and classification studies; 10 studies)	Most studies reported worse inhibitory control or higher variability in ABI^b^ or symptomatic mTBI^c^ compared with controls, and/or associations with symptom burden; findings were not uniform across paradigms and populations.	Very low	Serious risk of bias (mostly nonrandomized and incomplete reporting), serious inconsistency (heterogeneous paradigms or metrics), and serious imprecision (small samples and limited external validation).
Working memory or cognitive load effects (eg, n-back and dual-task paradigms)	Observational (case-control) with some classification analyses (3 studies)	Across cognitive-load paradigms, oculomotor impairment tended to be more evident under higher load; evidence is based on a few studies and selected samples.	Very low	Serious imprecision (few studies and small samples) and indirectness (task-specific paradigms and selected populations), with additional concerns about risk of bias in classification analyses.
Attention and predictive control (eg, smooth pursuit, visual tracking, predictive pursuit, and target blanking paradigms)	Observational and mixed designs (case-control and longitudinal or pre-post in a minority; 10 studies)	Several studies reported altered pursuit or visual tracking or predictive control in ABI groups, particularly under conditions that increase anticipatory demands; other studies reported smaller or null differences, limiting consistency.	Very low	Serious inconsistency (mixed results across paradigms), serious imprecision (small samples), and serious indirectness due to heterogeneity in paradigms, acquisition settings, and outcome definitions.
Visuospatial exploration or neglect detection (eg, free-viewing VR^d^ environments and spatial bias metrics)	Observational (1 study)	VR-based free-viewing eye tracking captured lateralized exploration patterns compatible with neglect phenotypes and may complement conventional tests; evidence is limited to a single study.	Very low	Very serious imprecision (single study) and indirectness (single setting or paradigm and limited generalizability).
Cognitive-communication and social cognition (eg, visual-world paradigms, AAC processing, and context interpretation)	Observational (case-control; 3 studies)	Eye-tracking measures suggested differences in efficiency of information processing and reduced integration of multimodal cues in ABI, but evidence is based on small, task-specific studies.	Very low	Serious imprecision (small samples), indirectness (task- and context-specific outcomes), and risk of bias related to selection and measurement.
Disorders of consciousness (DoC): command following or awareness detection using gaze-based paradigms	Noncomparative case series (2 studies)	A subset of patients achieved above-chance performance in gaze-selection tasks, suggesting potential to support bedside assessment; evidence is limited, and feasibility constraints were variably reported.	Very low	Very serious imprecision (small case series), serious risk of bias (noncomparative designs), and indirectness (variable arousal, assistive adaptations, and setting-specific implementation).
Diagnostic or classification performance of eye-tracking metrics (eg, discrimination of symptomatic mTBI vs controls and performance validity)	Diagnostic or classification studies (3 studies)	Eye-tracking–derived metrics showed discriminatory performance in specific paradigms, but thresholds, reference standards, and model validation procedures varied across studies.	Low	Risk of bias concerns (patient selection, index test conduct, and potential overfitting), inconsistency (different paradigms or thresholds), and imprecision (limited external validation).

a GRADE: Grading of Recommendations, Assessment, Development, and Evaluation.

b ABI: acquired brain injury.

c mTBI: mild traumatic brain injury.

d VR: virtual reality.

## Discussion

### Principal Findings

This systematic review synthesized 27 studies evaluating eye-tracking–derived metrics as a primary approach to quantify cognition or clinically relevant cognitive-communication processes after ABI. The evidence suggests that eye tracking can capture measurable differences in gaze control and visual attention that are plausibly related to executive inhibition, attentional prediction, and cognitive load. Findings were most consistently reported in chronic or persistent mTBI cohorts, while evidence for moderate-to-severe TBI, stroke, hypoxic-ischemic injury, and DoC was comparatively sparse and methodologically variable. Direct evidence of incremental validity over conventional neuropsychological testing was mixed and often indirect because comparator measures and analytic strategies varied across studies and were not consistently designed to test incremental value. Safety and feasibility reporting was inconsistent, limiting firm conclusions about tolerability and implementation at scale. Overall certainty of evidence was low across domains, reflecting small samples, heterogeneity of paradigms and outcome definitions, and limitations in internal and external validity.

### Interpretation and Clinical Implications

Eye-tracking outcomes can be interpreted at 2 complementary levels that should be kept conceptually distinct in clinical translation. Some outcomes primarily index sensorimotor oculomotor control, such as conjugate gaze stability, pursuit gain, and basic tracking precision. These can be clinically informative because visual sequelae are common after TBI, and ocular motor changes may reflect injury-related disruptions within the visual system or its supporting networks [87,88]. In contrast, paradigms such as antisaccade tasks, predictive pursuit under blanking or gap conditions, and cognitive load manipulations are more tightly coupled to executive inhibition, attentional prediction, and control processes. These paradigms can provide a process-level window into cognition, but their interpretability depends on careful alignment between task demands, analytic definitions, and the intended clinical construct. This distinction is important because a technically “abnormal” eye-movement pattern does not automatically imply impairment in a higher-order cognitive domain, and conversely, a patient may report cognitive symptoms despite relatively preserved performance on a specific oculomotor task.

From a biomarker perspective, the current literature supports a cautious stance. Eye-tracking metrics show promise as candidate behavioral biomarkers, but the present evidence base is not sufficiently standardized to support broad clinical adoption or claims of routine superiority over conventional assessment. Contemporary biomarker frameworks emphasize the need for rigorous validation, harmonized measurement pipelines, and clinically meaningful thresholds before a candidate measure can be used for diagnostic decision-making or treatment monitoring [89]. In our synthesis, diagnostic and classification analyses were reported in a subset of studies, but many relied on in-sample threshold selection, nonconsecutive sampling, or reference standards that were not directly aligned with cognitive-status classification. This pattern limits confidence in generalizability and reinforces the need for external validation and predefined analytic plans when classification performance is a central claim.

The clearest near-term clinical role for eye tracking may be as an adjunct rather than a replacement. In concussion and persistent symptom cohorts, eye tracking may be helpful when the goal is to quantify specific components of visual attention and control over short time windows, particularly when repeated measures are needed, and practice effects are a concern. Naturalistic and immersive implementations may also offer clinically meaningful advantages because they can capture cognitive-motor performance under conditions closer to real-world demands. VR-based neurocognitive testing that integrates eye tracking is an example of this direction, as it can link gaze behavior to task performance within ecologically relevant contexts [90]. At the same time, practical constraints remain nontrivial. Hardware costs, space requirements, calibration procedures, head stabilization needs, and trained personnel requirements can limit feasibility, particularly outside specialized centers. These barriers are consistent with broader concerns about implementation and scalability for technology-assisted assessment approaches in neurorehabilitation.

Specificity is another recurring concern that should be made explicit. Oculomotor metrics can be influenced by psychiatric symptoms, sleep disturbance, pain, medications, and demographic factors, all of which may be common after TBI and can co-vary with cognitive complaints. These influences do not invalidate eye tracking, but they constrain interpretation unless these covariates are measured and addressed analytically. In vulnerable or socially complex cohorts, comorbidities can be substantial and may contribute to both symptom burden and performance variability, underscoring the importance of careful phenotyping and confounder control in future studies [91,92]. Interpreting eye-tracking changes as cognition-specific without considering these influences risks overattribution and may inflate perceived diagnostic specificity.

### Translation to Neurorehabilitation and Future Directions

The results also inform the difference between demonstrated clinical utility and future potential. At present, most studies provide evidence of association, group discrimination, or feasibility rather than evidence that eye tracking improves clinical decisions or outcomes. Translational claims should therefore be framed as emerging. Still, several pathways appear promising. First, eye tracking may support precision rehabilitation by providing objective, high-frequency measures that can be tracked longitudinally and linked to individualized targets. This aligns with broader arguments that eye tracking can serve as a scalable behavioral signal of cognition in neurological conditions, provided that measurement is standardized and clinically interpretable [93]. Second, methodological work on quantifying and analyzing saccades, pursuit, and fixations offers a foundation for harmonizing metrics across devices and paradigms, which is necessary for multisite comparability and eventual normative datasets [94]. Third, broader clinical applications of eye tracking in medical practice suggest that eye tracking can be integrated into clinical workflows when the technical and interpretive challenges are addressed transparently [22].

A particularly important translational direction is the development of “digital biomarker” programs in which eye tracking is evaluated alongside complementary sources of evidence, including imaging, neurophysiology, and symptom trajectories. Reviews in other neurological conditions emphasize that eye-tracking metrics can be informative but require strong attention to measurement validity, confounding, and reproducibility to be credible as biomarkers [95]. Multimodal rehabilitation technologies, including VR and brain-computer interface concepts, may further enhance ecological validity and enable adaptive assessment and training paradigms, but these approaches require careful validation in ABI populations, especially regarding feasibility and tolerability in symptomatic cohorts [96,97]. In DoC, eye-tracking and HCI approaches may assist in detecting potential command following or residual cognition in select patients, but the literature remains small and sensitive to fluctuations in arousal and implementation constraints. These concerns align with broader discussions on recovery mechanisms and prognostication in DoC and reinforce the need for cautious interpretation of single-session performance metrics [98].

Future studies should prioritize methodological features that directly address the limitations identified in this review. Prospective registration, predefined primary outcomes, standardized reporting of calibration success and data loss, and device-specific acquisition parameters should become routine. External validation of classification thresholds and replication in broader etiologic subgroups are particularly important, given that the existing evidence is heavily weighted toward chronic mTBI and selected populations. In addition, longitudinal designs are needed to clarify whether eye-tracking metrics track recovery trajectories or identify subgroups at risk for persistent impairment. This is especially relevant given growing evidence that moderate-to-severe TBI can show chronic-stage cognitive decline patterns in some cohorts, which underscores the need for sensitive, repeatable measures over extended follow-up [99]. Finally, expansion beyond TBI is warranted. Eye tracking has been used to probe language processing and lexical integration, and emerging work suggests feasibility for remote or online eye tracking in language-related disorders, which may be informative for ABI cognitive-communication assessment if appropriately validated [100,101]. Similarly, additional evidence in prolonged DoC, stroke, and systems-level implementation could support a more balanced etiologic evidence base [102,103].

### Strengths and Limitations

This review provides a structured synthesis across cognitive domains and clinical use cases, incorporating design-appropriate risk-of-bias tools and an explicit certainty-of-evidence framework. The broad inclusion criteria allowed capture of a wide range of paradigms, including traditional oculomotor tasks, embedded cognitive-load assessments, VR paradigms, and HCI approaches. These strengths increase coverage and relevance across settings.

Several limitations constrain inference. First, the literature is highly heterogeneous in paradigms, outcome definitions, acquisition procedures, and analytic pipelines, which preclude quantitative pooling and limit the ability to estimate typical effect size ranges with confidence. Second, most studies were cross-sectional and underpowered, increasing vulnerability to imprecision and selective reporting. Third, the evidence base is dominated by chronic mTBI cohorts and selected populations, limiting generalizability to other ABI etiologies and to broader clinical settings. Fourth, safety and feasibility reporting was incomplete, which limits translation conclusions and obscures implementation barriers. Finally, diagnostic and classification claims require caution, as model development and thresholding were often performed within the same dataset, and reference standards varied, creating a risk of overestimated performance.

### Conclusion

Eye-tracking technology shows promising potential as an adjunctive approach for quantifying specific components of visual attention and oculomotor control that are plausibly linked to cognitive processes after ABI. The current evidence is most developed for paradigms involving inhibitory control, predictive tracking, and cognitive load in mTBI cohorts, while evidence for stroke, hypoxic-ischemic injury, and DoC remains preliminary and methodologically diverse. At present, the literature does not support broad claims that eye tracking routinely exceeds conventional neuropsychological assessment. Instead, the most defensible interpretation is that eye tracking may add clinically relevant information in paradigm- and population-specific contexts, particularly when standardized acquisition, transparent reporting, and appropriate confounder assessment are in place. Future research should focus on harmonized paradigms and reporting standards, external validation of classification thresholds, longitudinal designs, and explicit feasibility and safety reporting, with a goal of defining when and for whom eye tracking provides incremental clinical value within precision neurorehabilitation pathways.

## Supplementary material

10.2196/81276 Multimedia Appendix 1 Full search strategies.

10.2196/81276 Multimedia Appendix 2 Graphical abstract.

10.2196/81276 Checklist 1 PRISMA 2020 checklist.

## Abbreviations

ABI: acquired brain injury
DoC: disorders of consciousness
HCI: human-computer interface
mTBI: mild traumatic brain injury
NOS: Newcastle-Ottawa Scale
nTBI: nontraumatic brain injury
PRISMA: Preferred Reporting Items for Systematic Reviews and Meta-Analyses
QUADAS-2: Quality Assessment of Diagnostic Accuracy Studies 2
TBI: traumatic brain injury
VR: virtual reality
