# Supplementary material for: Eye-Tracking Technologies for Cognitive Assessment After Acquired Brain Injury: Systematic Review
Source: JMIR Rehabil Assist Technol. 2026 Jun 2;13:e81276. doi: 10.2196/81276 (PMC13229468; doi:10.2196/81276)
Supplement: Multimedia Appendix 1 [file rehab-v13-e81276-s001.docx]

**Review:** The Gaze That Guides: Eye-Tracking Technologies in Cognitive Assessment of Acquired Brain Injury

**Search dates:** Initial search 5 March 2025; updated 10 April 2025. Searches were run from database inception to 10 April 2025.

**Limits/filters:** English language where available; no publication date limits. Human/animal filters were not applied; animal studies were excluded during screening.

**Notes:** Syntax varies by database. The strategies below were used to capture (1) eye tracking/oculomotor measures, (2) cognition/cognitive assessment, and (3) acquired brain injury etiologies.

Field tags: [MeSH Terms], [Title/Abstract] where applicable.

**Search string**

("eye tracking technology"[MeSH Terms] OR ("eye tracking"[All Fields] AND "technology"[All Fields]) OR "eye tracking technology"[All Fields] OR ("eye"[All Fields] AND "tracking"[All Fields] AND "technology"[All Fields]) OR "eye tracking technology"[All Fields]) AND (("cognition"[MeSH Terms] OR "cognition"[All Fields] OR "cognitions"[All Fields] OR "cognitive"[All Fields] OR "cognitively"[All Fields] OR "cognitives"[All Fields]) AND ("assess"[All Fields] OR "assessed"[All Fields] OR "assessement"[All Fields] OR "assesses"[All Fields] OR "assessing"[All Fields] OR "assessment"[All Fields] OR "assessment s"[All Fields] OR "assessments"[All Fields])) AND ("brain injuries"[MeSH Terms] OR ("brain"[All Fields] AND "injuries"[All Fields]) OR "brain injuries"[All Fields] OR ("acquired"[All Fields] AND "brain"[All Fields] AND "injury"[All Fields]) OR "acquired brain injury"[All Fields]).
