# Supplementary figures and images for: Eye-Tracking Technologies for Cognitive Assessment After Acquired Brain Injury: Systematic Review
Source: JMIR Rehabil Assist Technol. 2026 Jun 2;13:e81276. doi: 10.2196/81276 (PMC13229468; doi:10.2196/81276)

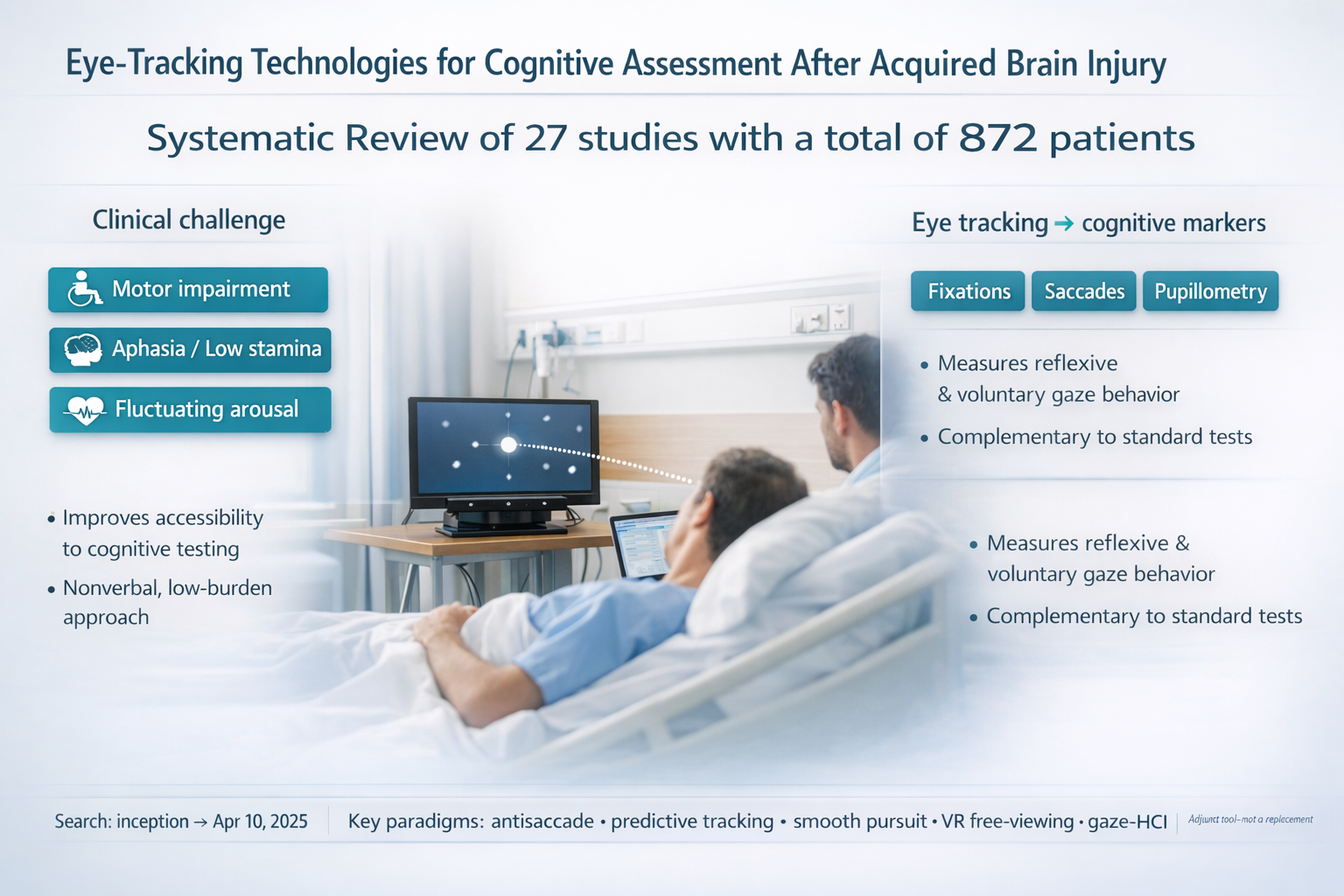

Supplement: Multimedia Appendix 2 [file rehab-v13-e81276-s002.png]
